# Exploring ComQXPA quorum-sensing diversity and biocontrol potential of *Bacillus* spp. isolates from tomato rhizoplane

**DOI:** 10.1111/1751-7915.12258

**Published:** 2015-03-10

**Authors:** A Oslizlo, P Stefanic, S Vatovec, S Beigot Glaser, M Rupnik, I Mandic-Mulec

**Affiliations:** 1 Department of Food Science and Technology, Biotechnical Faculty, University of LjubljanaLjubljana, Slovenia; 2 National Laboratory for Health, Environment and FoodMaribor, Slovenia; 3 Faculty of Medicine, University of MariborMaribor, Slovenia; 4 Centre of Excellence for Integrated Approaches in Chemistry and Biology of ProteinsLjubljana, Slovenia

## Abstract

*Bacillus subtilis* is a widespread and diverse bacterium t exhibits a remarkable intraspecific diversity of the ComQXPA quorum-sensing (QS) system. This manifests in the existence of distinct communication groups (pherotypes) that can efficiently communicate within a group, but not between groups. Similar QS diversity was also found in other bacterial species, and its ecological and evolutionary meaning is still being explored. Here we further address the ComQXPA QS diversity among isolates from the tomato rhizoplane, a natural habitat of *B*. *subtilis*, where these bacteria likely exist in their vegetative form. Because this QS system regulates production of anti-pathogenic and biofilm-inducing substances such as surfactins, knowledge on cell–cell communication of this bacterium within rhizoplane is also important from the biocontrol perspective. We confirm the presence of pherotype diversity within *B. subtilis* strains isolated from a rhizoplane of a single plant. We also show that *B. subtilis* rhizoplane isolates show a remarkable diversity of surfactin production and potential plant growth promoting traits. Finally, we discover that effects of surfactin deletion on biofilm formation can be strain specific and unexpected in the light of current knowledge on its role it this process.

## Introduction

It was already suggested by Darwin (1859) that intraspecific diversity increases the species adaptive potential to changing conditions, simply by making the population better prepared for the unexpected. Recently, this assumption was confirmed experimentally for various species, such as eelgrass where diversity within species contributes to its survival in fluctuating environments (Hughes and Stachowicz, 2004) and for *Pseudomonas aeruginosa* where strain diversity was shown to increase its stress resistance in biofilms (Boles *et al*., 2005). Moreover, experiments reveal that genetically uniform populations of bacteria can readily diverge, especially in structured environments that offer various niche opportunities (Rainey and Travisano, 1998; Poltak and Cooper, 2011).

*Bacillus subtilis*, ubiquitous and highly diverse Gram-positive bacterium, has been most often isolated in the form of heat resistant spores from soil and plant rhizosphere, as well as from other habitats such as aquatic systems, animal guts and various foods (Earl *et al*., 2008; Mandic-Mulec and Prosser, 2011). Its intraspecies diversity is reflected by high number of ecotypes (Koeppel *et al*., 2008; Stefanic *et al*., 2012; Kopac *et al*., 2014) and mirrored in diversification of distinct ‘communication’ groups or pherotypes (Tran *et al*., 2000; Tortosa *et al*., 2001; Ansaldi *et al*., 2002; Stefanic and Mandic-Mulec, 2009), which are defined as groups of bacteria that are able to communicate through signalling molecules (peptides) that elicit a response in strains sharing the same pherotype but not (or to significantly lesser extent) in those of a different pherotype (Ansaldi *et al*., 2002). Stefanic and colleagues (2012) proposed that pherotype diversity could be an adaptation to ecological diversity and showed that one pherotype dominates an ecotype with other pherotypes being present with lower frequency within an ecotype. Therefore, additional studies are needed to better understand the pherotype puzzle and its ecological meaning.

Diversification into pherotypes is coupled to striking polymorphisms of the ComQXPA quorum-sensing (QS) system (Tran *et al*., 2000; Ansaldi *et al*., 2002; Stefanic and Mandic-Mulec, 2009). It was shown that pherotypes can coexist in soil at a millimetre scale (Stefanic and Mandic-Mulec, 2009) and that the communication diversification is under Darwinian selection (Ansaldi and Dubnau, 2004) and is present also in other *Bacillus* species that encode the *comQXPA* homologues loci (Dogsa *et al*., 2014). Still, it is not clear how this diversification is manifested in other parts of the genome and how it is adaptive for the species. The ComQXPA lingual system operates the QS (Fuqua *et al*., 1994), a process where secreted signalling molecules (ComX) accumulate to critical concentration and by activation of cognate receptors (ComP), trigger the expression of target genes. More precisely, ComX signal is initially synthesized as 55 amino acid-long prepeptide that is processed by ComQ and secreted from the cells (Magnuson *et al*., 1994; Ansaldi *et al*., 2002; Schneider *et al*., 2002). Signal production by *B. subtilis* serves as a negative feedback mechanism which modulates QS response of producing cells (Oslizlo *et al*., 2014). When ComX accumulates, it activates ComP receptor, which then phosphorylates response regulator ComA, and this one in turn modulates the transcription of many genes (Weinrauch *et al*., 1996; Comella and Grossman, 2005). Microarray studies revealed that *srfAA-D* operon encoding the surfactin synthetase (Nakano *et al*., 1988) accounts for the most affected target by the ComQXPA regulon (Comella and Grossman, 2005).

Surfactin is a powerful lipopeptide biosurfactant and an antibiotic that acts against many bacteria and fungi, including plant pathogens like *Pseudomonas syringae* (Bais *et al*., 2004). In addition, surfactin by inducing a potassium leakage in the cells (Lopez *et al*., 2009) indirectly serves as a signal that triggers biofilm formation, which is essential for colonization of roots (Beauregard *et al*., 2013; Zeriouh *et al*., 2013) and protection of plants against pathogens (Bais *et al*., 2004; Chen *et al*., 2013; Zeriouh *et al*., 2013). In fact, *B. subtilis* is regarded to be a PGPR (plant growth-promoting rhizobacterium) and has been well known for its biocontrol potential (Barea *et al*., 2005; Berg, 2009; van Elsas and Mandic-Mulec, 2013). It was even proposed that the vegetative form of this species is normally associated with plant roots and that soil is predominantly inhabited by its dormant spores (Norris and Wolf, 1961). If this holds true, it is of major importance to complement our current knowledge on the communication diversity of *B. subtilis* isolates that live on plant root surfaces (rhizoplane), especially because the diverse ComQXPA system controls the expression of biocontrol agents, like surfactin (Nakano *et al*., 1988; Zeriouh *et al*., 2013). PGPR are widely accepted as ecofriendly alternatives to chemical pesticides, and they have been in commercial use for several years (Nakkeeran *et al*., 2005). Knowledge on QS diversity in rhizoplane and on how this diversity manifests in QS-regulated traits could then contribute to optimal design of PGPR-based formulations.

Most work on *B. subtilis* ecology has been performed by studying spores isolated from soil (Mandic-Mulec and Prosser, 2011). Here, we use a set of *B. subtilis* isolates and close relatives isolated from tomato rhizoplane to address the genetic and functional diversity of spore formers. Our aim was to examine whether different QS pherotypes of *B. subtilis* vegetative cells can coexist within the rhizoplane of a single plant. We further addressed whether being a member of a certain pherotype in the rhizoplane manifests in similar expression of known ComQXPA-regulated biocontrol properties like production of surfactin or biofilm formation. In addition, we compare direct plant growth promotion and potential PGP traits between isolates derived from a single plant or within a pherotype. We find that *B. subtilis* strains living on roots of a single plant carry diverse QS pherotypes and are highly diverse with respect to their biocontrol potential. The strains show differences in biofilm formation, surfactin production and other PGPR traits and most interestingly, behave differently after silencing of the *srfA* operon. We discuss what such intraspecies diversity could mean for the bacteria, for the plant and for biocontrol by rhizoplane communities.

## Results

### Isolation and characterization of *Bacillus* spp. isolates

**Fig 1:**
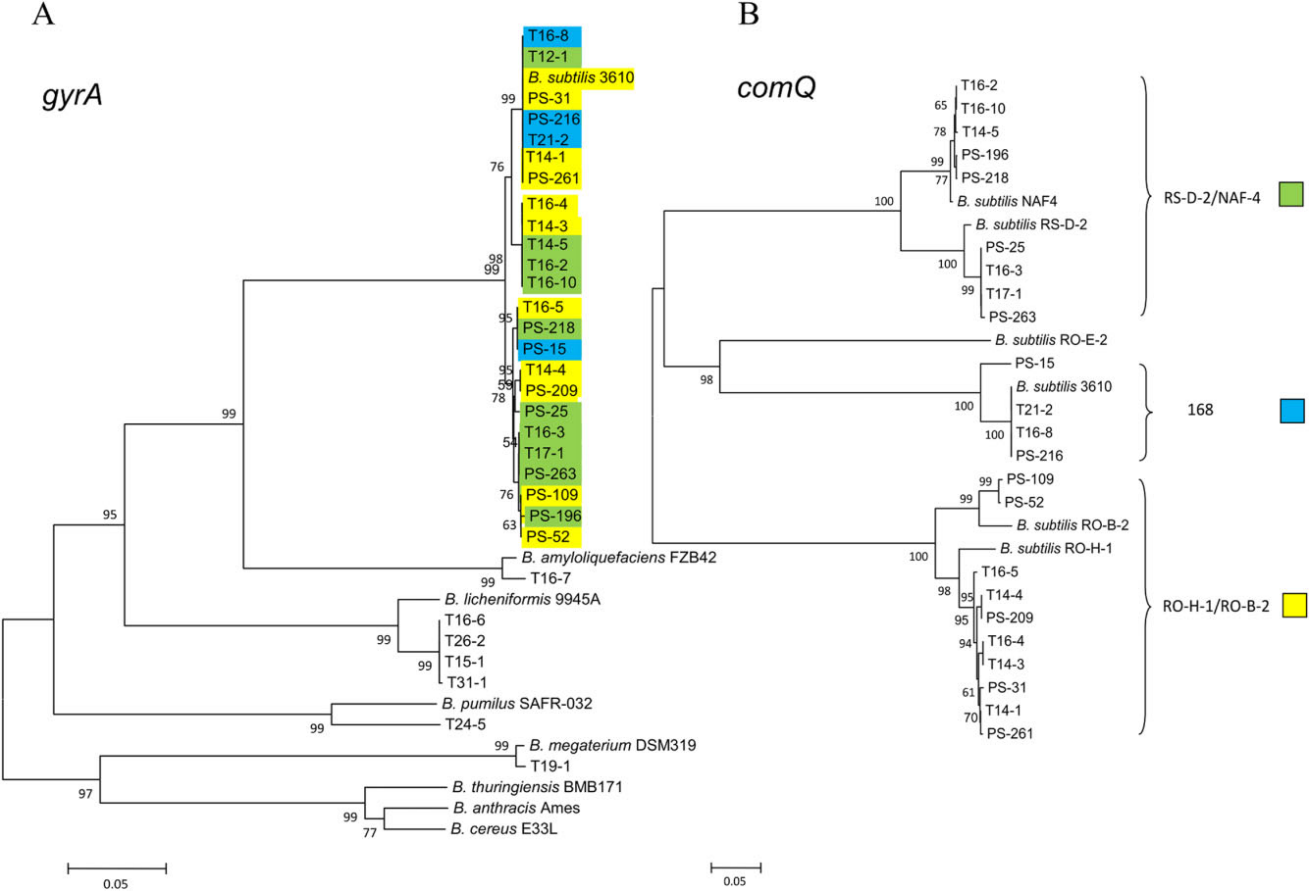
Minimum evolution trees based on (A) partial gyrA nucleotide sequences (610 bp) and (B) partial comQ sequences (701 bp).A. Strains representing pherotype 168, RO-H-1/RO-B-2 and RS-D-2/NAF-4 are higlighted in blue, yellow and green respectively.B. Pherotypes with this colour code are indicated on the right side of the corresponding clades.

Rhizoplanes of 21 different tomato plants grown at two different locations (A and B; see *Experimental procedures*) were screened for *Bacillus subtilis*-like colonies. Strains (*n* = 20), phenotypically resembling colonies of *B. subtilis*, were obtained from 10 out of 21 tomato plants. Sequencing of 16S rRNA confirmed that they all belong to the *Bacillus* genus; however, due to high percentage of sequence similarity, we could not determine the species affiliation for all isolates based on 16S rRNA sequences. We therefore further identified the strains on the basis of the *gyrA* gene, which can be used as an alternative phylogenetic marker because of higher rates of molecular evolution as compared with 16S rRNA (Chun and Bae, 2000). This approach allowed us to identify *B. subtilis* (13 strains), *B. licheniformis* (4), *B. amyloliquefaciens* (1), *B. pumilu*s (1) and *B. megaterium* (1) (Fig. 1A).

This approach was preferred over MIDI (Microbial Indentification System) Similarity index, which provided taxonomic identification only for 17 out of 20 strains (Table S1). *Bacillus subtilis* strains were isolated from 5 out of 21 plants suggesting heterogeneity among viable rhizoplane *Bacilli* and all *B. subtilis* strains listed in Table S1 were used for further experiments. The *B. subtilis gyrA* sequences were highly conserved (∼ 99% sequence identity), but still some sequence subclusters could be identified on the *gyrA* ME (minimum evolution) similarity tree (Fig. 1A). These subclusters included *B. subtilis* strains isolated from one plant, different plants and even from plants at different locations. Moreover, *gyrA* sequences of rhizoplane isolates were at minimal genetic distances (∼ 100% sequence identity) with *gyrA* of *B. subtilis* isolated from the Sava riverbank soil, Slovenia (Stefanic and Mandic-Mulec, 2009), and no habitat-dependent clustering was observed (Fig. 1A).

### Polymorphism of *comQXP* locus

High polymorphism within *comQXP* genes was previously confirmed for soil *B. subtilis* isolates (Ansaldi *et al*., 2002; Stefanic and Mandic-Mulec, 2009). In this study, we find a similar pattern for rhizoplane isolates (Fig. 1B). The *comQ* sequences were highly polymorphic and fall into three clusters: 168 (blue), RS-D-2/NAF4 (green) and RO-H-1/RO-B-2 (yellow), with only 65–70% identity at the nucleotide level between clusters (Fig. 1B). Each of the three clusters, depicted by the ME similarity tree of 12 rhizoplanes and few representative *comQ* sequences of the soil isolates (Fig. 1B), contained sequences from rhizoplane and soil bacteria. Also isolates derived from one plant (plant 16: strains T16) were randomly distributed along the tree, and each cluster contained at least one T16 isolate.

Diversity of *comQ* was also found within sequence similarity clusters as noticed in our previous study (Stefanic and Mandic-Mulec, 2009). Rhizoplane *comQ* sequences within the RS-D-2/NAF4 cluster split into two distinct subclusters, with ∼ 85% *comQ* sequence identity between and 100% within the subclusters. However, within RO-H-1/RO-B-2 and 168 clusters, the *comQ* sequence diversity of the rhizoplane isolates was low, with 99% or 100% similarity respectively. This could be due to significantly lower number of isolates analysed here as compared with the previous studies involving soil isolates. As expected, ME trees based on *gyrA* and *comQ* were not congruent and over 30% *comQ* divergence was found among strains carrying clonal *gyrA* sequences (Fig. 1).

### Specificity of the *comQXP* QS loci

All *Bacillus spp.* isolates were tested for their activation of QS response of six tester strains representing different pherotypes. Tester strains carrying a P*srfA-lacZ* reporter fusion were grown in conditioned media of the rhizoplane isolates and then tested for β-galactosidase activity representing transcriptional response of *srfA* to ComX present in the condition medium. Specific producer strains were used as positive controls.

**Fig 2:**
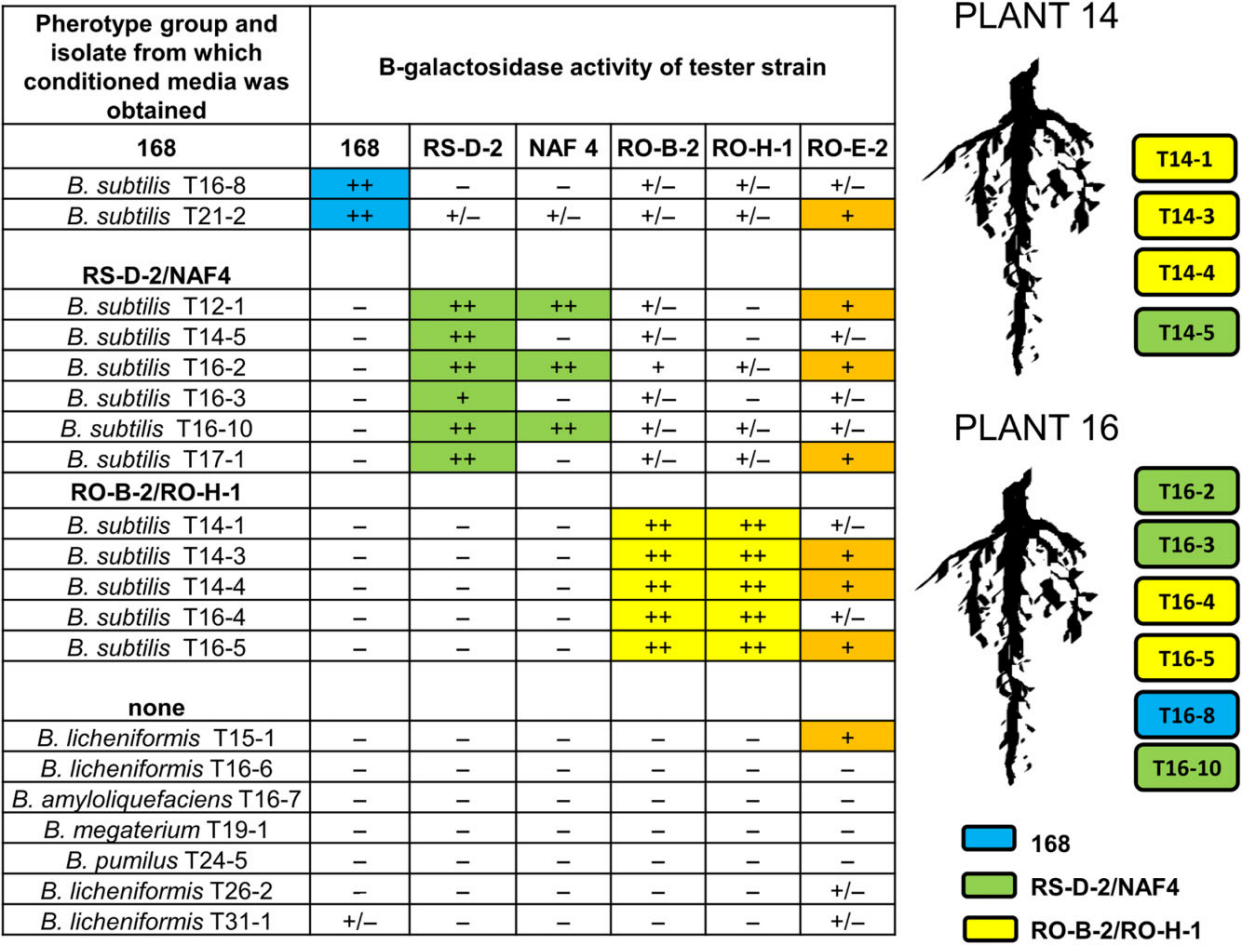
Specific activation of the QS response was measured using tester strains able to detect one of the four previously determined pherotypes through activation of the srfA-lacZ reporter. The testers were specific for the pherotype 168 (blue), the pherotype RS-D-2 /NAF4 (green); the pherotype RO-B-2/RO-H-1 (yellow); and the pherotype RO-E-2 (orange). Rhizosphere isolates were grown in CM and conditioned media were sampled 1 h after entry into the stationary phase (T1). Tester strains were then inoculated (1:50) into conditioned medium mixed with an equal volume of fresh CM medium, grown for 16 h and assayed for β-galactosidase activity as indicated in *Experimental procedures*. Symbols: ++, strong response, similar to positive control; +, moderate response, approximately 50% of the positive-control response; +/−, weak but reproducible response; −, no activation. On the right: schematic drawing representing pherotype diversity of rhizoplane isolates from plant 14 and plant 16.

On the basis of strong and moderate activation responses, 15 out of 20 strains could be classified to three distinct pherotypes (Fig. 2). These pherotypes were consistent with clustering of *comQ* sequences (Fig. 1B), and we concluded that two strains (T16-8 and T21-2) belong to the pherotype 168; five strains (T14-1, T14-3, T14-3, T16-4 and T16-5) to the pherotype RO-H-1/ RO-B-2; and six strains (T12-1, T14.5, T16-2, T16-3, T16-10 and T17-1) were confirmed as the pherotype RSD-2/NAF-4. Interestingly, only three isolates (T12-1, T16-2 and T16-10) out of six induced a strong response of the NAF4 tester strain, suggesting a possible split of the pherotype and continuous evolution of the QS genes as previously indicated for soil microscale isolates (Stefanic and Mandic-Mulec, 2009). All strains that induced strong response of the tester (marked as ‘++’) belonged to *B. subtilis* species. Some cross-talk of *B. subtilis* resulting in a moderate or very low response (‘+’ or ‘+ /−’) with testers outside the primary pherotype could be observed; however, the cross-talk never induced response as strong as the pherotype specific communication (Fig. 2). Finally, the *B. licheniformis* T15-1 conditioned medium induced moderate response of *B. subtilis* RO-E-2 tester, suggesting the presence of cross species communication. The results together with phylogenetic analysis confirmed that *B. subtilis* strains of distinct pherotypes can be isolated from the rhizoplane of a single plant supporting a ubiquity of the pherotype diversity (Fig. 2).

### Diversity of potential biocontrol properties within *B. subtilis* pherotypes and plants

As both surfactin production (Comella and Grossman, 2005) and indirectly also biofilm formation (Lopez *et al*., 2009) are under ComQXPA QS control, we examined the variability of these traits within the rhizoplane collection of *B. subtilis* isolates. We were particularly interested whether this diversity is evident also within a pherotype. We used the *B. subtilis* BD2833 strain (Tortosa *et al*., 2001), which is deficient in surfactin production and biofilm formation (BD 2883 derives from 168 described in McLoon *et al*., 2011) as a negative control. In addition, the *B. subtilis* GB03 strain was used as a positive control for biofilm formation (Beauregard *et al*., 2013).

**Fig 3:**
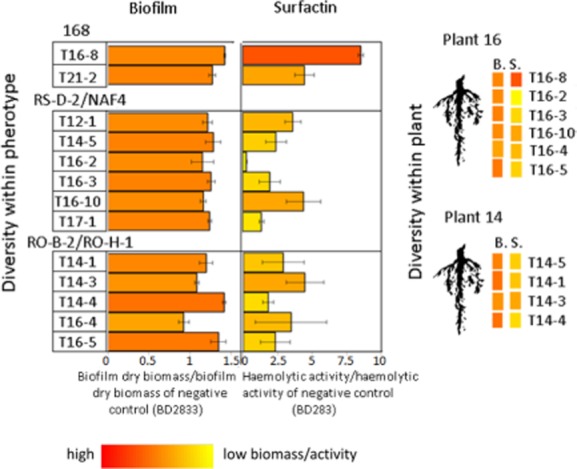
Biofilm biomass and surfactin activity of *B. subtilis* isolates from tomato rhizoplane. Biofilms were harvested and their dry mass was determined. The dry mass of each biofilm was then divided by the dry mass of biofilm formed by the negative control BD2833. Conditioned media produced during biofilms growth were filter-sterilized, and the presence of biosurfactants was determined by heamolytic test. Strain BD2833 which is deficient in surfactin production was used as negative control. Percent of haemolysis obtained for each conditioned medium was divided by the value obtained for negative control. Columms on the graph indicating the diversity of the response are marked with RGB intensities that directly correspond to quantitative values measured for each trait (as shown below the table). Data represent average of three independent replicates with SE (standard error) indicated. The RGB colours also indicate the diversity of biofilm dry biomass (B.) and haemolytic activity (S.) at the plant level (on the right).

**Fig 4:**
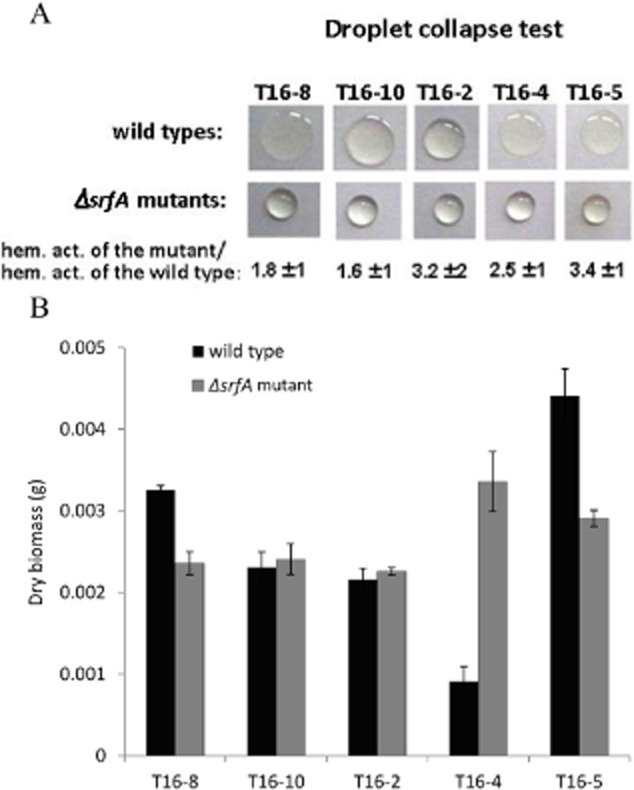
(A) Conditioned media (30 μ droplets) of wild isolates T16-8, T16-2, T16-10, T16-4 and T16-5 (first raw) and of isogenic ΔsrfA mutants (second raw) from pellicle cultures grown in MSN medium at 28°C were sampled after 48 h. The numbers below the droplet pictures represent percent of haemolytic activity measured in conditioned medium of isogenic ΔsrfA mutants as compared with their ancestor wild type strains. (B) Biofilms were harvested after 48 h of incubation, and their dry mass was determined. Data represent average of three independent replicates (independent experiments).

We observed that all but one *B. subtilis* isolates produced pellicle biofilms with higher biomass as compared with negative control and that three isolates (T16-8, T14-4 and T16-5) produced larger biofilm biomass as compared with the positive control (GB03 strain that exhibited 1.19. ± 0.3 value) (Fig. 3, Fig. S1). Within one pherotype, strains differed in both tested traits: biofilm formation and surfactin production (Fig. 3, Fig. S1). For example, within the pherotype RO-H-1/RO-B-2, one strain formed a copious biofilm and another was comparable with a negative control (Fig. 3). The diversity of surfactin production was also very pronounced, and within each pherotype we could find very strong biosurfactant producers (up to eight times more as compared with the control) but also strains that showed very weak surfactin activity (measured by haemolytic activity) or no such activity at all (Fig. 3). Similar diversity could be observed when strains were grouped in respect to the plant they were isolated from (Fig. 4A). For example, each plant (plant 14 and plant 16) contained a very strong surfactant producer (T14-3 and T16-8 respectively), a moderate producer or even a non-producer (T16-4) (Fig. 3). In order to confirm that the haemolytic assay measured surfactin-specific activity of the conditioned media produced by rhizoplane isolates, we managed to inactivate the *srfA* gene in five isolates: T16-8, T16-10, T16-4, T16-5 and T16-2 by incorporation of the *ΔsrfA* mutation (see *Experimental procedures*) using a standard transformation protocol applied for naturally competent *B. subtilis*. We successfully transformed five out of eight strains presumably due to differences in transformation efficiency of natural isolates (data not shown). We compared the haemolytic activity of the mutants with their ancestors. All 5 *ΔsrfA* mutants showed over 95% decrease of haemolytic activity (Fig. 4A). Therefore, haemolytic activity of wild rhizoplane isolates was surfactin dependent. Moreover, the drop collapse test (Jain *et al*., 1991), which is an alternative, qualitative method of surfactin detection, correlated with the haemolytic assay (Fig. 4A).

Next we tested the effects of surfactin deletion on biofilm biomass production. Interestingly, not all strains were affected equally. In the T16-8 and T16-5 strains, surfactin deficiency decreased biofilm biomass by 28% (*P* < 0.02) and 34% (*P* < 0.02), respectively, while the T16-2 and T16-10 mutants formed biofilms in biomass comparable with the wild-type ancestors. Surprisingly, the T16-4-*ΔsrfA* mutant had 3.5-fold (*P* < 0.02) larger biomass than the parental strain (Fig. 4B). Therefore, the regulatory role of surfactin in biofilm formation might be strikingly different among natural isolates of *B. subtilis*, and it will be interesting to identify genetic differences behind this observation in the future.

### PGPR properties within *B. subtilis* pherotypes

**Fig 5:**
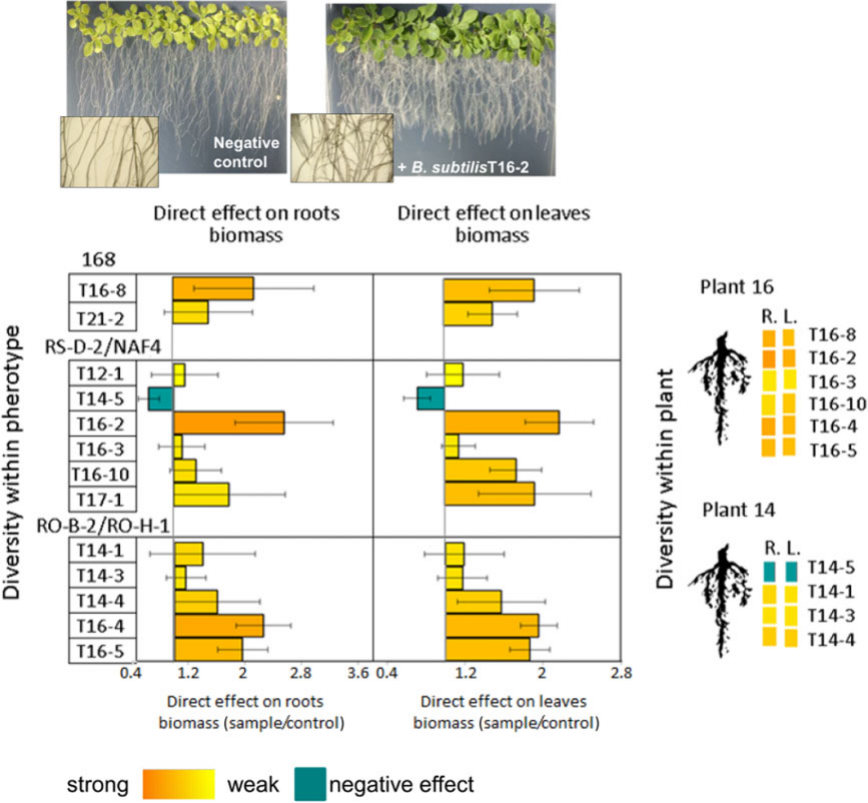
Influence of *B. subtilis* isolates from tomato rhizoplane on *A. thaliana* roots and leaves biomass. Plants were grown for 14 days in app. 5 cm distance from the bacterial inoculums. Positive effect of *B. subtilis* isolate presence on growth of the model plant is shown on the pictures above. Final biomass of roots and leaves was divided by biomass of control plants (roots and leaves) that were grown on sterile medium. To better show the diversity in the response within each indicated pherotype (168, RS-D-2 /NAF4, RO-B-2/RO-H-1), columns on the graph were marked with RGB intensities that directly correspond to quantitative values measured for each trait (as shown below the graphs). Data represent average of the three independent replicates with SE indicated. The colours representing effect on roots biomass (R.) and leaves biomass (L.) were also used to demonstrate the diversity at the plant level (on the right).

It is known that bacteria can directly promote the growth of plant by various secretions (Kloepper *et al*., 1980; López-Bucio *et al*., 2007). We therefore tested whether secreted molecules of the rhizoplane isolates can influence the growth of plant roots and leaves of the model plant *Arabidopsis thaliana*. Bacteria were inoculated on 0.2× MS (Murashige and Skoog) solid medium (see *Experimental procedures*) app. 5 cm away from the *A. thaliana* seedlings, and the plant biomass versus control (no bacteria inoculated) was measured after 10 days (Fig. 5). Isolates sharing a pherotype or being derived from the rhizoplane of a single plant (Fig. 5) had different effects on roots and leaves biomass. For example, two strains of the same pherotype RS-D-2/NAF4, namely T16-2 and T16-4, increased the biomass of roots (*P* < 0.06 and *P* < 0.002, respectively) and leaves (*P* < 0.03 and *P* < 0.006, respectively) twofold, while the strain T16-3 of the same pherotype had no effect on the plant biomass (Fig. 5).

Similar diversity was found among isolates from one plant, with plant 16 isolates giving very strong PGP effect or no effect; and isolates from plant 14 showing weak positive effects, no effects, or even negative effects on plant biomass (Fig. 5).

**Fig 6:**
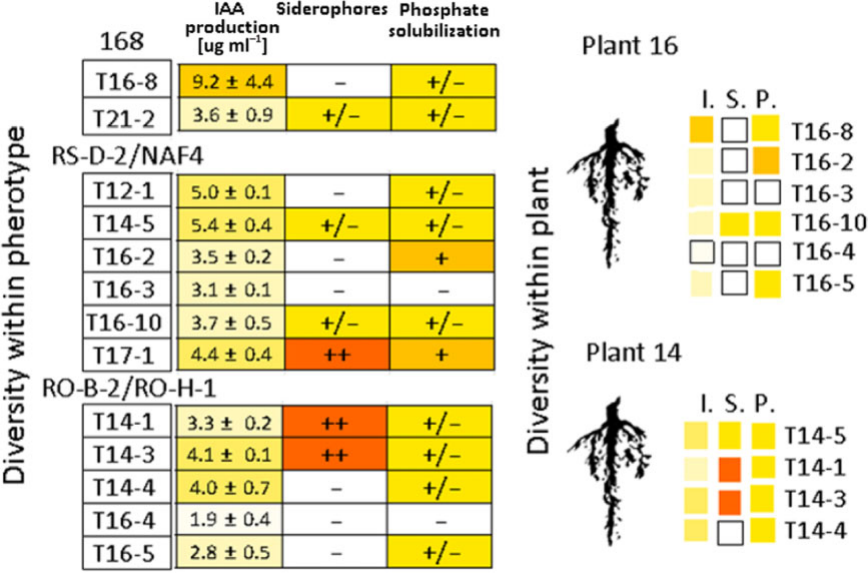
Comparison of potential PGP traits: IAA production, siderophores production and phosphate solubilization. Symbols: ++, strong effect, +, moderate effect (significantly lower as compared to maximal effect observed); +/−, weak but reproducible effect, –, no effect. The RGB intensity was used to better show the diversity of the traits. The RGB colours are used to indicate the diversity of PGP traits: IAA concentration (I.), siderophores (S.) and phosphate solubilization (P.) at the plant level (on the right).

In addition, we analysed three phenotypic traits that can influence plant growth: production of indole-3-acetic acid (IAA) (López-Bucio *et al*., 2007) or siderophores (Kloepper *et al*., 1980) and the ability to solubilize phosphate (Molla *et al*., 1984). These properties were also variable within a pherotype, and there was no correlation between plant growth promotion measured directly and these traits. For example, strain T16-4, which had strong positive effect on plant biomass, was negative in all PGP traits tested (IAA, siderophores and phosphate solubilization) (Fig. 6). Similarly, strains T16-10 and T16-7 were positive in two out of three PGP traits but did not promote the growth of roots (Fig. 6). This indicates that direct effects on plant growth cannot be easily predicted only by testing the established PGP traits.

In addition to PGP bacteria, we also found inhibitors of plant growth: T14-5 (*B. subtilis*, pherotype RS-D-2/NAF-4) (Fig. 5) and T31-1 (*B. licheniformis*) (Table S2) induced 30% decrease in root's (*P* < 0.08 and *P* < 0.05, respectively) and leave's (*P* < 0.09 and *P* < 0.06 respectively) biomass. Additionally, we isolated an interesting PGP candidate from plant 19, *B. megaterium* T19-1, which produced approximately five times higher concentration of IAA (25 μg ml^−1^) compared with other strains, showed very strong production of siderophores and also strongly (twofold) promoted the growth of roots (*P* < .004) and leaves (*P* < .002) of *A. thaliana* (Table S2).

## Discussion

*Bacillus subtilis* strains that persist in soil are classified to three to four distinct QS groups – pherotypes (Ansaldi *et al*., 2002; Stefanic and Mandic-Mulec, 2009). As plant rhizosphere was proposed the main habitat of *B. subtilis* vegetative existence (Norris and Wolf, 1961), we asked here whether the pherotype diversity is also found among rhizoplane isolates.

We confirmed that different QS groups (pherotypes) can coexist on roots of a single plant. This was shown by *comQ* sequence analysis and by specific induction of QS response. Despite low number of *B. subtilis* isolates per plant (6 isolates from plant 16 and 4 isolates from plant 14), we found a comparable diversity, manifested in three pherotypes, which was previously observed for soil millimetre scale (Stefanic and Mandic-Mulec, 2009), confirming that *comQXP* diversity is widespread and easy to find. This again brings up the fundamental question on adaptive role of this diversity, which is also found in other gram-positive bacteria, including *Staphylococcus aureus*, *Streptococcus pneumoniae* (Pozzi *et al*., 1996; Ji *et al*., 1997; Whatmore *et al*., 1999; Carrolo *et al*., 2014) and *B. cereus* (Slamti and Lerecius, 2005). Stefanic and colleagues (2012) found that most but not all *B. subtilis* strains of the same ecotype belong to the same pherotype and proposed that pherotypes may at least in part be under ecological selection. Rhizoplane strains were not analysed for ecotype association, but their high phenotypic diversity (e.g. variability in surfactin production and other biocontrol properties) suggests that many of them may be ecologically distinct. Interestingly, isogenic strains of *S. pneumoniae* of distinct pherotypes differed in their ability to form biofilms, possibly because of pherotype-dependent strength of QS signalling (Carrolo *et al*., 2014). Here we found no correlation between QS type (pherotype) and the expression of a QS-regulated trait – surfactin production. This is in contrast to observation by Carrolo and colleagues (2014), who noticed such interrelationship, but it should be stressed that they used isogenic strains, which only differed in pherotypes. We used wild isolates of different genetic backgrounds that may additionally influence the surfactin synthesis and secretion and dominate over pherotype association.

Strong variability in surfactin production among rhizoplane isolates, even among isolates of a single plant, may also influence social life of this species. Because surfactin is secreted and probably shared between neighbouring *B. subtilis* strains, it may, under certain conditions, serve as a public good (West *et al*., 2007). Differences in surfactin production could also result in disproportions in metabolic investment and fitness among strains (Oslizlo *et al*., 2014), allowing weak surfactin producers to benefit from strong producers when plant pathogen invades and needs to be opposed. Coexistence of social and less social phenotypes was previously found in neighbouring strains of *Myxococcus xanthus* (Kraemer and Velicer, 2014) or *P. aeruginosa* (Wilder *et al*., 2011). In case of *M. xanthus* it was shown that ‘social’ strains can promote the persistence of less social isolates without negative effects on the social's fitness, but sometimes when highly abundant, the less social strains can decrease fitness of the whole community (Kraemer and Velicer, 2014). It was recently suggested that spatial segregation facilitates the evolution of cooperation in *B. subtilis* biofilms (van Gestel *et al*., 2014). Therefore, high spatial segregation of different pherotypes might help to stabilize cooperative traits and public goods production, like surfactin. Consequently, selection for cooperation might lead to assortment and diversity of pherotypes even at small distances. In addition, it was recently discovered that *B. subtilis* colonizes hyphae of *Aspergilus niger* where surfactin expression is downregulated by these fungi (Benoit *et al*., 2014). Could then the decreased level of surfactin secretion in certain *B. subtilis* strains (like T16-2 orT14-4) be an adaptation to peaceful coexistence with fungi, which are also common rhizoplane inhabitants?

It is not known how presence of poor surfactin producers influences the performance of the whole *B. subtilis* community in fighting plant pathogens, and it is an interesting problem of sociomicrobiology that could be addressed in the future. It is also worth noting that in certain isolates (like T16-10 or T16-2), the presence of surfactin showed no influence on biofilm biomass and in others (like T16-8 and T16-5) even exerted a negative influence on biofilm biomass. It is known that surfactin serves as a paracrine signal for biofilm formation (Lopez *et al*., 2009). Our results, however, indicate that surfactin role in biofilm formation may be more complex and strain specific. This result also further supports previous assumptions about high genetic and phenotypic diversity of strains sharing a pherotype. Moreover, because surfactin is required for root colonization (Bais *et al*., 2004; Chen *et al*., 2013), it remains to be tested how the observed differences in surfactin production translate into root-colonization abilities of the *B. subtilis* isolates. Also, it will be interesting to address whether the *ΔsrfA* mutants (T16-2; T16-5: T16-5: T16-8 and T16-10) lose the ability to colonize the plant roots, despite different effects of *srfA* deletion on pellicle biofilms.

We observed only moderate diversity at the level of biofilm formation. Although we did not look into biofilm-related gene expression, our results are in line with previous data showing that genetic diversity within *B. subtilis* is especially high with respect to antibiotic-related genes (like surfactin) and low with respect to biofilm-related genes (Earl *et al*., 2008). The reason for the later may be associated with attached growth being essential for rhizocompetence and probably represents a competitive advantage in rhizoplane.

While our strain isolation strategy does not allow us to speculate on the original spatial distribution among the *Bacillus* spp. isolates on roots, the results confirm that different pherotypes reside on roots of a single plant.

Stefanic and colleagues (2012) proposed that ratios of pherotypes continuously cycle in nature because of induction of costly products released by high-frequency pherotype and temporary advantage of the low-frequency pherotype. Therefore, the diversity of pherotypes could be naturally selected by means of social conflict based on release of costly products (Eldar, 2011; Stefanic *et al*., 2012). However, because members of one pherotype dramatically differ in QS-regulated biocontrol traits, direct effects on plant growth and other potential PGP behaviours (IAA secretion, production of siderophores or phosphate solubilization), pherotype diversity would not threaten the biocontrol function of the *Bacillus* community – eventually each pherotype contains a strong surfactin producers, or/and direct plant growth promoters. Therefore, the diversity of pherotypes on plant roots may promote coexistence of different strains and thus positively influence the biocontol potential of this species.

It was previously shown that plant growth promotion by *B. subtilis* depends on production of volatile 2,3-butanediol (Ryu *et al*., 2003). Because we found no correlation between PGP effects and analysed secretions (IAA, siderophores and phosphatases), it is possible that volatile molecules could influence the growth of a model plant in our experiment; however, this awaits further studies.

The idea to use microbes as biocontrol agents emerged many years ago, and many studies screening for PGP properties of rhizosphere and rhizoplane isolates were performed. Some strains were patented and its commercial use is steadily increasing (Maheshwari, 2011). Although it is known that diverse community of microbes determines the plant health, PGPR-based preparations are still based on monocultures (Maheshwari, 2011). There were several successful attempts of applying multispecies formulations (Raupach & Kloepper 1998; Singh *et al*., 1999), but sometimes, simply because of interspecies competition, the effects can be just opposite to the expectations (Chiarini *et al*., 1998; de Boer *et al*., 1999). In fact, in terms of sharing a niche, one should rather expect a competition instead of a synergy between microbial species (Foster and Bell, 2012). It is therefore important to bring more attention to intraspecies genetic and phenotypic diversity in the rhizosphere and rhizoplane, where next to strong indirect competition, more cooperation, especially within a pherotype, would be predicted. Testing biocontrol properties or plant growth-promotion effects of mixed *B. subtilis* communities should be the next step to answer this question.

We show here that *B. subtilis* residing on roots differ in QS pherotypes, potential PGPR traits and the ability to influence the growth of *A. thaliana*. We believe this study opens new interesting questions about the role of strain diversity in arms race between *B. subtilis* and plant pathogens, or in interactions with host plant. We also believe that applying diverse strains of one genus or species carrying diverse biocontrol properties should be considered as an alternative to non-monoculture-based biocontrol agents design.

## Experimental procedures

### Bacterial strains

Strains used in the study are listed in Table 1 and Table 2. In Table 1, we listed isolates from the tomato rhizoplane and also three control strains: 6051, GB03 and FZB42, which were previously reported to show PGP properties (Bais *et al*., 2004; Idris *et al*., 2004; Xie *et al*., 2009). Tomato rhizoplane isolates are marked with T and the following number corresponds with the plant so that, for example, strains T14-1, T14-3 and T14-4 were isolated from the rhizoplane of the same plant (T14). Table 2 includes engineered *B. subtilis* strains that were used to test the specificity of rhizoplane and riverbank isolates in activating the QS response in six tester strains, representing four currently recognized pherotypes. In addition Table 2 includes five *ΔsrfA* mutants that were obtained by transforming eight randomly chosen *B. subtilis* rhizoplane T-isolates with chromosomal DNA isolated from the OKB120 strain (Nakano *et al*., 1988; Vollenbroich *et al*., 1996). To induce natural competence, *B. subtilis* T-isolates were grown in competence medium (CM) (Albano *et al*., 1987) for 6 h. Briefly, 0.5 ml of early stationary phase cultures were mixed with 1 μg of OKB120 chromosomal DNA suspension and incubated for 30 min at 37°C with vigorous shaking. Next, the cultures were supplemented with 0.5 ml of Luria–Bertani (LB) medium and incubated for additional 60 min. Transformants were selected on agar plates containing lincomycin (12.5 μg ml^−1^) and erythromycin (0.5 μg ml^−1^).

**Table 1:** *Bacillus spp*. isolates used in this study

Strain	Location	Plant	*Bacillus* species	Reference
T12-1	A	12	*B. subtilis*	This work
T14-1	A	14	*B. subtilis*	This work
T14-3	A	14	*B. subtilis*	This work
T14-4	A	14	*B. subtilis*	This work
T14-5	A	14	*B. subtilis*	This work
T16-2	A	16	*B. subtilis*	This work
T16-3	A	16	*B. subtilis*	This work
T16-4	A	16	*B. subtilis*	This work
T16-5	A	16	*B. subtilis*	This work
T16-8	A	16	*B. subtilis*	This work
T16-10	A	16	*B. subtilis*	This work
T15-1	A	15	*B. licheniformis*	This work
T16-6	A	16	*B. licheniformis*	This work
T16-7	A	16	*B. amyloliquefaciens*	This work
T17-1	B	17	*B. subtilis*	This work
T21-2	B	21	*B. subtilis*	This work
T19-1	B	19	*B. megaterium*	This work
T24-5	B	24	*B. pumilus*	This work
T26-2	B	26	*B. licheniformis*	This work
T31-1	B	31	*B. licheniformis*	This work
GB03	na	na	*B. subtilis*	BGSC 3A37
FZB42	na	na	*B. amyloliquefaciens*	BGSC 10A6
6051	na	na	*B. subtilis*	ATCC DSM 10

na, does not apply; strains were obtained from the Bacillu Genetic Stock Center (BGSC) and from The Leibniz Institute DSMZ.

**Table 2:** Engineered *B. subtilis* strains used in this study

Producer strains		
BD2833	*his leu met srfA-lacZ* (*tet*)	Tortosa and colleagues (2001)
BD2913	*his met srfA-lacZ* (*tet*) *amyE*::*xylR Pxyl-comK* (*ery*) (*comQ comX comP* replaced by genes from *B. mojavensis* RO-H-1)	Tortosa and colleagues (2001)
BD2915	*his met srfA-lacZ* (*tet*) *amyE*::*xylR Pxyl-comK* (*ery*) (*comQ comX comP* replaced by genes from *B. subtilis* natto NAF4)	Tortosa and colleagues (2001)
BD2936	*his met srfA-lacZ* (*tet*) *amyE*::*xylR Pxyl-comK* (*cat*) (*comQ comX comP* replaced by genes from *B. mojavensis* RO-B-2)	Tortosa and colleagues (2001)
BD2940	*his leu met srfA-lacZ* (*tet*) *amyE*::*xylR Pxyl-comK* (*cat*) (*comQ comX comP* replaced by genes from *B. subtilis* RO-E-2)	Ansaldi and colleagues (2002)
BD2949	*his leu met srfA-lacZ* (*tet*) *amyE*::*xylR Pxyl-comK* (*cat*) (*comQ comX comP* replaced by genes from *B. subtilis* RS-D-2)	Ansaldi and colleagues (2002)
**Tester strains**		
BD2876	*his leu met srfA-lacZ* (*tet*) *comQ*::Km	Tortosa and colleagues (2001)
BD2877	*his leu met srfA-lacZ* (*tet*) (*comQ*::*phl comX comP* replaced by genes from *B. subtilis* natto NAF4)	Tortosa and colleagues (2001)
BD2962	*his met srfA-lacZ* (*tet*) *amyE*::*xylR Pxyl-comK* (*ery*) (*comQ*::pED345 *comX comP* replaced by genes from *B. mojavensis* RO-H-1)	Tortosa and colleagues (2001)
BD2983	*his leu met srfA-lacZ* (*tet*) *amyE*::*xylR Pxyl-comK* (*cat*) (*comQ*::pED345 *comX comP* replaced by genes from *B. mojavensis* RO-B-2)	Ansaldi and colleagues (2002)
BD3019	*his leu met srfA-lacZ* (*tet*) *amyE*::*xylR Pxyl-comK* (*cat*) (*comQ*::pED375 *comX comP* replaced by genes from *B. subtilis* RS-D-2)	Ansaldi and colleagues (2002)
BD3020	*his leu met srfA-lacZ* (*tet*) *amyE*::*xylR Pxyl-comK* (*cat*) (*comQ*::pED375 *comX comP* replaced by genes from *B. subtilis* RO-E-2)	Ansaldi and colleagues (2002)
***ΔsrfA* mutants**		
T16-8ΔsrfA	*srfA::Tn917 (mls)*^a^	This work
T16-10ΔsrfA	*srfA::Tn917 (mls)*^a^	This work
T16-2ΔsrfA	*srfA::Tn917 (mls)*^a^	This work
T16-4ΔsrfA	*srfA::Tn917 (mls)*^a^	This work
T16-5ΔsrfA	*srfA::Tn917 (mls)*^a^	This work

a srfA::Tn917 (mls) insertion was derived from genomic DNA of OKB120 strain (Nakano *et al*., 1988; Vollenbroich *et al*., 1996).

### Isolation and characterization of T-strains from tomato rhizoplane

In October 2011, 21 tomato plants from two home vegetable gardens (A and B) located 60 km apart in north-west Slovenia were sampled. Bulk soil was carefully removed from the roots, and samples were transferred to sterile plastic bags. Roots were then washed with sterile saline solution. Six pieces of 1 cm long roots were collected in 1 ml sterile saline solution and homogenized with pestles for 3 min. The suspension was heat shocked (Brain Heart Infusion) at 70°C for 10 min, and serial dilutions were plated on BHI (Brain Heart Infusion) agar. After 48 or 72 h of incubation at room temperature, *Bacillus*-like colonies were subcultured. Crude DNA was isolated from pure culture by boiling one loop of culture in 100 μl of 5% Chelex® (BIO-RAD). To confirm, *B. subtilis*-specific polymerase chain reaction (PCR) was carried out using the primers gyrAF (5′-CAGTCAGGAAATGCGTACGTCCTT-3′) and gyrAR1 (5′-CAATGAGAGTATCCGTTGTGCGTC-3′). For additional analysis, the Sherlock Microbial Identification System (Agilent Technologies) was used.

### DNA amplification and sequencing

DNA from strains was extracted using classical phenol-chloroform method. The 16S rRNA genes were amplified by PCR with primers 27F (5′-AGAGTTTGATCMTGGCTCAG-3′) and 1406R (5′-ACGGGCGGTGTGTRCAA-3′) (Nubel *et al*., 1996). The 16S rRNA genes were sequenced using the reverse primer 1406R (5′ACGGGCGGTGTGTRCAA-3′). The *gyrA* genes were amplified by PCR with primers gyrAR6 (5′-3′) and gyrAF5 (5′-3′), and *gyrA* gene was sequenced using the reverse primer gyrAR6 (5′-3′). The *comQ* genes were amplified by PCR with primers Uni-comQ1 (5′-GGGAGGGGGGAAGTCGTTATTG-3′) and P1 (5′-AAGAACCGAATCGTGGAGATCGCG-3′) (Tortosa *et al*., 2001). The *comQXP* locus was sequenced with the forward primer Uni-comQ1 (5′-GGGAGGGGGGAAGTCGTTATTG-3′); PCR products were sequenced by Macrogen (Seoul, Korea). Standard BLAST algorithm (standard nucleotide BLAST available at http://blast.ncbi.nlm.nih.gov/Blast.cgi) using default settings was used for sequence analysis/provisional identification of *Bacillus* spp.

### Phylogenetic analyses

Phylogenetic analyses were conducted using mega version 4 (Tamura *et al*., 2007) for neighbour-joining and ME analyses using Tamura–Kumar model of evolution with heterogeneous patterns among lineages and gamma-distributed rates among sites. The evolutionary history was inferred using the ME method (Rzhetsky and Nei, 1992). The bootstrap consensus tree inferred from 1000 replicates (Falsenstein, 1985) was taken to represent the evolutionary history of the taxa analysed. Branches corresponding to partitions reproduced in less than 50% bootstrap replicates were collapsed. The percentage of replicate trees in which the associated taxa clustered together in the bootstrap test (1000 replicates) were shown next to the branches (Felsenstein, 1985). The tree was drawn to scale, with branch lengths in the same units as those of the evolutionary distances used to infer the phylogenetic tree. For *gyrA* sequences and *comQ* sequences, the evolutionary distances were computed using the Tamura 3-parameter method (Tamura, 1992) and maximum composite likelihood method (Tamura *et al*., 2004), respectively, and are in the units of the number of base substitutions per site. The ME tree was searched using the close-neighbour-interchange algorithm (Nei and Kumar, 2000) at a search level of 1. The neighbour-joining algorithm (Saitou and Nei, 1987) was used to generate the initial tree. Codon positions included were 1st+2nd+3rd+ non-coding. All positions containing gaps and missing data were eliminated from the dataset (complete deletion option). For *gyrA* and *comQ-*based analysis, there were a total of 822 and 827 positions in the final dataset respectively.

### β-Galactosidase assay

β-Galactosidase was assayed using a Multiscan Spectrum Microplate Reader (Thermo Scientific). The absorbance at 420 nm was measured at 30°C immediately after the addition of ortho-nitrophenyl-β-galactoside substrate. Tester strains were incubated with shaking for 16 h at 28°C in 100 μl fresh CM medium and 100 μl of conditioned media produced by rhizoplane and riverbank isolates and harvested in T1. After the incubation, cells were centrifuged (4 °C; 1,800 × g) and re-suspended in 200 μl Z-buffer with 5.6% (vol/vol) β-mercaptoethanol before adding 10 μl toluene and incubating the cultures on ice for 30 min. The plate was then warmed to 30°C, 50 μl ortho-nitro-phenyl-β-galactoside substrate was added and the absorbance (420 nm) was immediately determined at 30°C.

### Biofilm formation assay

Assay was performed using MSN (minimal salts nitrogen) medium (5 mM potassium phosphate buffer pH 7, 0.1 M Mops pH 7, 2 mM MgCl2, 0.05 mM MnCl2, 1 μM ZnCl2, 2 μM thiamine, 700 μM CaCl2, 0.2% NH4Cl) supplemented with 0.5% pectin as performed by Beauregard and colleagues (2013). Strains were grown overnight in LB, and suspensions were inoculated (2%) into MSN pectin media distributed in 10 ml Petri dishes and grown for 48 h at 28°C. Pellicles were harvested, dried and weighted. Conditioned media were sampled for biosurfactant assay.

### Biosurfactant antibiotics production

Cells were grown as described above (biofilm formation assay), and conditioned media were collected. Biosurfactants activity was measured using haemolytic assay (Moran *et al*., 2002). Bovine red blood cells (RBC) were washed two times with isotonic buffer (140 mM NaCl and 20 mM Tris pH 7.4) and once with 0.9% NaCl. The RBC were then resuspended in 0.9% NaCl to optical density 0.7. Dilution series of conditioned media were prepared and 100 μl of supernatant fraction, 30 μl of 96% ethanol and 100 μl of RBC were mixed on microtitre plate. Optical density (λ = 650 nm) was measured immediately after addition of RBC and after 15 min of incubation at room temperature. The percentage of decrease in optical density that was in linear correlation to sample dilution value was transformed into percentage of haemolysis and divided by corresponding biofilm dry biomass.

In addition, drop collapse test was performed (Jain *et al*., 1991). The drops of 30 μl of conditioned media were transferred to smooth parafilm surface, and image was taken after 5 min of incubation in room temperature. Flatten droplets indicated presence of biosurfactant.

### Plant growth promotion assay

Arabidopsis ecotype Col-0 seeds were surface sterilized using 2% sodium hypochlorite solution. Briefly, seeds were incubated in 2% sodium hypochlorite with mixing on an orbital mixer for 20 min and then washed five times with sterile distilled water.

Seeds were germinated and grown on agar plates containing MS medium (Murashige and Skoog basal salts mixture; Sigma) (2.2 g l^−1^) supplemented with 1% sucrose. After 3 days of incubation at 4°C, plates were transferred to plant growth chamber (photoperiod of 16 h of light, 8 h of darkness, light intensity, constant temperature of 24°C) and placed vertically at an angle of 65 degrees. After 10 days, homogenous 1 cm long seedlings were selected for growth promoting experiments.

Bacterial strains were grown in LB medium until late exponential phase, cells were washed twice with 0.9% NaCl and 50 μl suspensions were inoculated on MS agar plates in a line, approximately 2 cm from the bottom of the plate. Plates were incubated overnight in 37°C. Next, 16 1 cm-long Col-0 seedlings were transplanted to the MS plates, approximately 5 cm away from the bacterial line and arranged similarly as described by López-Bucio and colleagues (2007). After 10 days of incubation in plant growth chamber, the seedlings were removed from the agar, the roots were washed with distilled water, separated from the leaves and measured with a ruler. Roots and leafs from each MS plate were collected and weighted before and after drying. Roots and leaves mass obtained from bacteria-inoculated plates was compared with control, where seedlings were grown on sterile plates.

### IAA production

*Bacillus* spp. isolates were grown in LB medium supplemented with L-tryptophan (Sigma, T0254) (1 mg ml^−1^ final concentration) for 48 h at 28°C with shaking 200 r.p.m. Supernatants were collected and IAA production was determined with the use of iron and perchloric acid according to modified method of Solon and Weber (1950). Briefly FeCl3-HClO_4_ reagent (1.0 ml of 0.5 M FeCl3, 50 ml 35% HClO_4_) was mixed with the culture supernatant in 1:2 ratio and incubated for 15 min. Next 5 μl of orthophosphortic acid was added for reaction enhancement and absorbance at λ = 510 nm was determined. IAA concentration was calculated from a standard curve prepared using commercial IAA (Sigma, I3750).

### Siderophore production

Bacterial strains were grown overnight on solid media (Schwyn and Neilands, 1987) prepared as follows: 100 ml of Minimal Media 9 (MM9) stock solution (15 g KH_2_PO_4_, 25 g NaCl, 50 g NH_4_Cl dissolved in 500 ml of ddH_2_O) was mixed with 750 ml of MiliQ and 15 g of agar. After autoclaving and cooling to 50°C, the medium was supplemented with 30 ml of 10% sterile casamino acid solution (BD, 223050) and 10 ml of sterile 20% glucose solution. MM9-based media with *Bacillus* growth were each overlaid by 10 ml of the following medium: 60.5 mg chrome azurol S, 72.9 mg hexadecyltrimetyl ammonium bromide, 30.24 g piperazine-1,4-bis(2-ethanesulfonic acid) and1 mMFeCl3·6H2O in 10 mMHCl 10 ml, 9 g of agar per 1 l of MiliQ. After a maximum period of 15 min, a change in colour from blue to purple around the colonies indicated the siderophore producers (Pérez-Miranda *et al*., 2007). Qualitative estimation of siderophore production was performed as follows: – negative (no orange halo), from + /− to ++, positive.

### Phosphate solubilization

The ability of the strains to solubilize inaccessible phosphate was determined using Pikovskaya agar (Pikovskaya, 1948). Cells were grown overnight in LB medium, next they were washed twice with 0.9% NaCl and re-suspended in 0.9% NaCl to produce equal cell densities among all the isolates. Solutions were inoculated on the agar plates and incubated in 37°C for 7 days. The size of halo (zone of solubilization) around the bacterial colony indicated phosphate solubilizing abilities of each strain. Qualitative estimation of phosphate solubilization was performed as follows: – negative (no halo), from + /− to ++, positive.

## Supporting Information

**Fig. S1.** Images of pellicle biofilms were taken after 48 h of pellicles growth in MSN medium (see *Experimental procedures*) at 28°C. Strain BD2833 which is a derivative of laboratory strain IS75 was used as negative control, and strain GB03 which is known biopesticide was used as positive control. **Table S1.** The MIDI Sherlock microbial identification system was used to identify *Bacillus* spp. isolates based on the fatty acid methyl ester profile of the bacteria. The program ‘Sherlock microbial identification system’ was used to compare the fatty acid methyl ester profiles of the bacteria and strains were identified based on similarity index. Last column indicates the identification based on *gyrA* sequence identity. **Table S2.** Data on other *Bacillus* spp. rhizoplane isolates: effects on roots and leaves biomass and IAA production – determined quantitatively; and qualitative estimation of siderophores production and phosphate solubilization. Symbols: ++, strong effect, +, moderate effect (significantly lower as compared to maximal effect observed); +/−, weak but reproducible effect, –, no effect. RGB (Red, Green, Blue) intensity was used to better show the diversity.
