# Supplementary material for: Exploring ComQXPA quorum-sensing diversity and biocontrol potential of Bacillus spp. isolates from tomato rhizoplane
Source: Microb Biotechnol. 2015 Mar 10;8(3):527–40. doi: 10.1111/1751-7915.12258 (PMC4408185; doi:10.1111/1751-7915.12258)
Supplement: Supplementary file 1 [file mbt20008-0527-sd1.zip › MBT2_12258-supp-0001-Supplementary figure 1.docx]

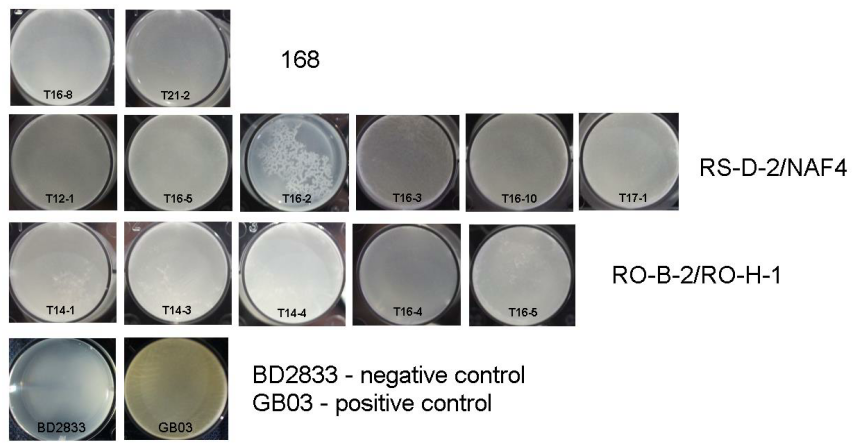


Supplementary figure 1. Images of pellicle biofilms were taken after 48 hours of pellicles growth in MSN medium (see methods) at 28°C. Strain BD2833 which is a derivative of laboratory strain IS75 was used as negative control, and strain GB03 which is known biopesticide was used as positive control.
