# Supplementary material for: Exploring ComQXPA quorum-sensing diversity and biocontrol potential of Bacillus spp. isolates from tomato rhizoplane
Source: Microb Biotechnol. 2015 Mar 10;8(3):527–40. doi: 10.1111/1751-7915.12258 (PMC4408185; doi:10.1111/1751-7915.12258)
Supplement: Supplementary file 1 [file mbt20008-0527-sd1.zip › MBT2_12258-supp-0002-Supplementary table 1.docx]

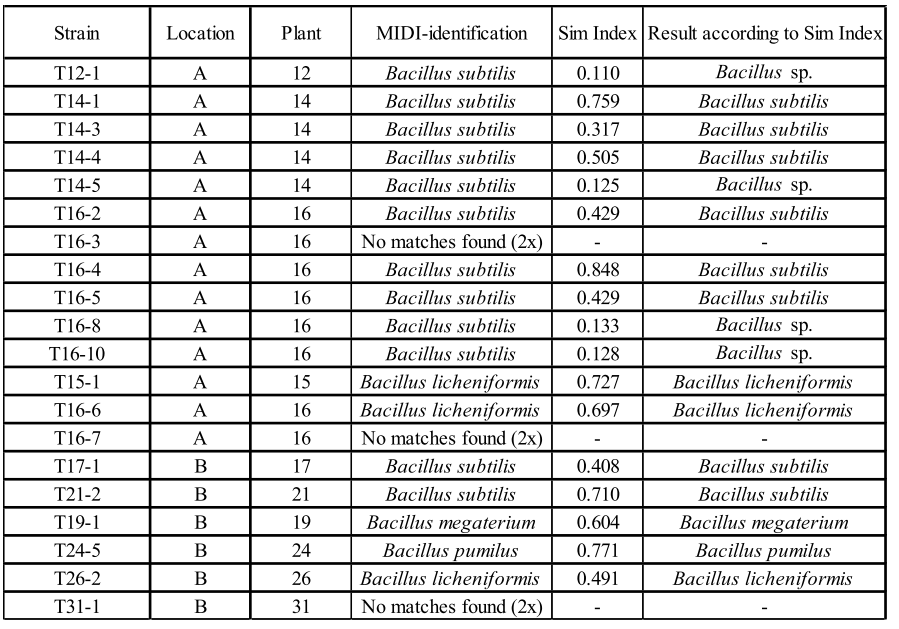


Supplementary table 1. The MIDI Sherlock microbial identification system was used to identify *Bacillus* spp. isolates based on the fatty acid methyl ester profile of the bacteria. The program “Sherlock microbial identification system” was used to compare the fatty acid methyl ester profiles of the bacteria and strains were identified based on similarity index.  Last column indicates the identification based on *gyrA* sequence identity.
