# Supplementary material for: Exploring ComQXPA quorum-sensing diversity and biocontrol potential of Bacillus spp. isolates from tomato rhizoplane
Source: Microb Biotechnol. 2015 Mar 10;8(3):527–40. doi: 10.1111/1751-7915.12258 (PMC4408185; doi:10.1111/1751-7915.12258)
Supplement: Supplementary file 1 [file mbt20008-0527-sd1.zip › MBT2_12258-supp-0003-Supplementary table 2.docx]

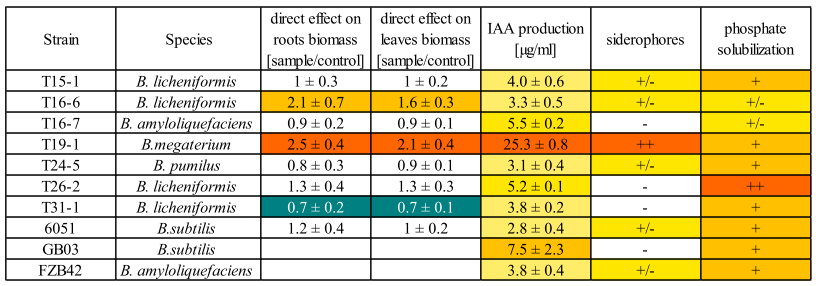


Supplementary table 2. Data on other *Bacillus* spp. rhizoplane isolates: effects on roots and leaves biomass and indole-3-acetic acid production (IAA) – determined quantitatively; and qualitative estimation of siderophores production and phosphate solubilization. Symbols: ++, strong effect, +, moderate effect (significantly lower as compared to maximal effect observed); +/−, weak but reproducible effect, - no effect. RGB intensity was used to better show the diversity.
